# A Recurrent Neural Network Model for Predicting Activated Partial Thromboplastin Time After Treatment With Heparin: Retrospective Study

**DOI:** 10.2196/39187

**Published:** 2022-10-13

**Authors:** Sebastian Daniel Boie, Lilian Jo Engelhardt, Nicolas Coenen, Niklas Giesa, Kerstin Rubarth, Mario Menk, Felix Balzer

**Affiliations:** 1 Charité – Universitätsmedizin Berlin, corporate member of Freie Universität Berlin and Humboldt-Universität zu Berlin Institute of Medical Informatics Berlin Germany; 2 Charité – Universitätsmedizin Berlin, corporate member of Freie Universität Berlin and Humboldt-Universität zu Berlin Department of Anesthesiology and Intensive Care Medicine Berlin Germany; 3 Charité – Universitätsmedizin Berlin, corporate member of Freie Universität Berlin and Humboldt-Universität zu Berlin Institute of Biometry and Clinical Epidemiology Berlin Germany

**Keywords:** machine learning, health care, recurrent neural network, heparin, activated partial thromboplastin time (aPTT), deep learning, ICU, critical care

## Abstract

**Background:**

Anticoagulation therapy with heparin is a frequent treatment in intensive care units and is monitored by activated partial thromboplastin clotting time (aPTT). It has been demonstrated that reaching an established anticoagulation target within 24 hours is associated with favorable outcomes. However, patients respond to heparin differently and reaching the anticoagulation target can be challenging. Machine learning algorithms may potentially support clinicians with improved dosing recommendations.

**Objective:**

This study evaluates a range of machine learning algorithms on their capability of predicting the patients’ response to heparin treatment. In this analysis, we apply, for the first time, a model that considers time series.

**Methods:**

We extracted patient demographics, laboratory values, dialysis and extracorporeal membrane oxygenation treatments, and scores from the hospital information system. We predicted the numerical values of aPTT laboratory values 24 hours after continuous heparin infusion and evaluated 7 different machine learning models. The best-performing model was compared to recently published models on a classification task. We considered all data before and within the first 12 hours of continuous heparin infusion as features and predicted the aPTT value after 24 hours.

**Results:**

The distribution of aPTT in our cohort of 5926 hospital admissions was highly skewed. Most patients showed aPTT values below 75 s, while some outliers showed much higher aPTT values. A recurrent neural network that consumes a time series of features showed the highest performance on the test set.

**Conclusions:**

A recurrent neural network that uses time series of features instead of only static and aggregated features showed the highest performance in predicting aPTT after heparin treatment.

## Introduction

Thromboembolic complications are associated with increased mortality [1,2]. Risk factors for deep venous thrombosis and pulmonary embolism include, for example, immobility, malignancy, higher age, and a history of thromboembolism [3,4]. Anticoagulation by drugs is applied either prophylactically to prevent thromboembolism [5] or therapeutically to treat existing thromboembolic complications, which reduces mortality [6].

In perioperative normal care wards, prophylactic and therapeutic anticoagulation is frequently performed subcutaneously by low–molecular weight heparins [5]. In the perioperative setting, prophylactic anticoagulation is indicated in patients with intermediate or high risk for thromboembolism. This includes, for example, most trauma surgeries, elective orthopedic surgeries with consecutive immobility of the lower limbs, and major abdominal or thoracic surgery, particularly in the presence of malignant and inflammatory processes [5].

In critical illness, the risk for venous thromboembolism is increased in almost all patients due to the combination of general risk factors related to chronic disease and intensive care unit (ICU)–associated risk factors, including sedation, immobility, or central venous catheters [7]. In intensive care, prophylactic or therapeutic anticoagulation is regularly applied intravenously by continuous unfractionated heparin, particularly during renal failure or hemodynamic instability [8]. The short half-life of the anticoagulant and the possibility of antagonizing heparin with protamine are advantages of unfractionated heparin in these vulnerable patients [9]. However, poor controllability is an issue. Consequently, overdosing with hemorrhagic or underdosing with thrombotic complications may occur [10]. Hence, therapeutic unfractionated heparin application requires monitoring. The dosing of unfractionated heparin is performed by determination of activated partial thromboplastin time (aPTT) in patients' blood [11]. Based on older studies, the pursued aPTT target is approximately a 1.5 to 2.5-times prolongation of the reference clotting time [11-13] although individual targets are usually defined. Achieving the aPTT target within 24 hours has been associated with increased survival in patients with pulmonary embolism [6]. However, due to patient- and disease-related variations, achieving the aPTT target within 24 hours is challenging.

Nowadays, big data sets are generated by digital patient data management systems in ICU routine. Machine learning (ML) approaches that include individual information from large data sets may help to predict aPTT at an earlier stage than can routine blood sampling. Previous results of applying ML to predict aPTT show great promise [14-17]. Some authors [16,17] consider the numerical value of aPTT and consequently the prediction of aPTT as a regression task. We prefer the prediction of the numerical value since it makes no assumption of the aPTT target range. However, most recent literature on similar-sized data sets consider aPTT after heparin treatment as a multiclass prediction with 3 distinct ranges (subtherapeutic, therapeutic, or supratherapeutic) [14,15,18].

In previous model comparison studies [15,16,18], it has been demonstrated that artificial neural networks show the highest performance on aPTT prediction tasks.

Recently, a systematic review of ML approaches on predicting aPTT after heparin administration highlighted that still multiple innovations are required before ML-assisted heparin dosing is ready for clinical practice [19].

We compared multiple ML models on our patient cohort and are, to our knowledge, the first to apply a recurrent neural network model that takes the dynamics of variables in the form of time series into account. At the outset of the study, we specified inclusion criteria that resulted in 5926 distinct hospital admissions. On this cohort, we trained and evaluated multiple ML models on the aPTT prediction task. To allow comparison of the recurrent neural network model with previously published models [14,15,18], we subsequently used our model in a classification setup.

The aim of this analysis is to evaluate whether ML models can accurately predict subsequent aPTT measurements well (12 hours) in advance. In the future, data-driven approaches could help clinicians to adjust heparin dosing to improve time in the target range aPTT after 24 hours.

## Methods

### Data Selection Criteria

The database system for surgical and intensive care patients at Charité – Universitätsmedizin Berlin (Charité) was first adopted in 2012 and over time rolled out to all ICUs. Since we extracted data in November 2021, we considered a time period from 2012 to October 31, 2021. We selected patients and hospital admissions that satisfied the following inclusion criteria: at least 18 years old at the beginning of treatment, received a minimum of 1000 IU of heparin, received some of the heparin as continuous infusion, had at least a single aPTT measurement after 12 hours and before 36 hours after the intravenous treatment commenced, and had weight and height documented (within reasonable limits: height between 25 cm to 250 cm, weight between 3 kg to 300 kg).

### Ethics Approval

Ethics approval for this study was obtained by the Charité ethics committee (vote #EA4/241/21).

### Feature Selection and Prediction Targets

We extracted patient characteristics (age, gender, height, weight), laboratory values (aPTT, bilirubin, C-reactive protein, creatinine, quick value, platelet count), whether patients received dialysis or a form of extracorporeal membrane oxygenation (ECMO) treatment, and routinely collected scores (therapeutic intervention scoring system 10 [TISS-10], simplified acute physiology score [SAPS-II], sequential organ failure assessment [SOFA], acute physiology and chronic health evaluation II [APACHE II]) from the hospital information system. Furthermore, we extracted the time of the start and end of each heparin dosing, concentration, and administration rate. Heparin can be administered as a bolus or as a continuous infusion. All data were restricted to the time period 7 days prior to treatment to 36 hours after treatment started.

Our goal was to predict the aPTT 24 hours after initiation of continuous heparin treatment. However, not all patients had a laboratory measurement exactly 24 hours after the treatment with heparin. Thus, any aPTT measurement between 12 to 36 hours after heparin treatment began was accepted as the prediction target. In case multiple values were recorded between 12 hours and 36 hours, we chose the value that was closest to 24 hours after continuous treatment started. Consequently, only values that were taken before or within 12 hours after continuous heparin treatment commenced were available as features for the model (including any aPTT measurement in that time frame). Hospital stays were left aligned, and the start of the continuous intravenous heparin delivery corresponded to time zero.

### Handling of Missing Data

The data we used for our study were collected during routine care and were not of uniform quality across all hospital admissions. A typical problem when using retrospective data for ML is missing observations [20-22]. This problem is exacerbated for the recurrent neural network, as it expects an input for every feature every 2 hours.

The static values of gender, age, height, and weight had no missing values and were replicated for every timestamp. The one-hot–encoded variables, including ECMO treatment, dialysis, bolus delivery of heparin, and continuous delivery of heparin, were set to 0 if no other value was recorded for a given timestamp. Other features (eg, laboratory measurements and scores) were filled in a 2-step process as follows: (1) If a previous value was recorded within 7 days prior to continuous heparin treatment, those values were forward filled; (2) Any still missing values were replaced by the mean across the training population.

Only using the above 2-step procedure discards information about which measurement is from the patient at the given timestamp. Since it has been shown that the missing pattern can be informative [23], we included an “indicator” variable for each variable filled in the 2-step process that is 1 if the value was measured at the given timestamp and 0 if it was imputed.

Together with the indicator variables, each model sees 35 different input variables.

The recurrent neural network, thus, may see time series between *t* = –168 (7 days prior to continuous heparin delivery) to *t*= 12. In general, however, patients’ time series are not of the same length.

### Models and Variable Encoding

The input data consisted of numerical and categorical variables. Categorical variables (gender, ECMO treatment, dialysis treatment, continuous heparin administration, bolus heparin administration) were one-hot encoded. Each option for a categorical variable resulted in 1 input dimension that could either be 1 or 0. One-hot–encoded variables were not further scaled and were directly used as input features.

Other numerical variables were standardized before being fed into the model. Mean and SD were estimated only on the training data set.

We compared 6 models that take a single value per feature and 1 model that takes the entire time series of features. Some features were constant over the course of treatment (age, gender, height, and weight), while the other features changed frequently. Models that take a single value per feature received the last-observed value before the 12-hour cutoff. The recurrent neural network received time series, resampled to 2-hour intervals, for each feature. If multiple measurements were taken within 2 hours, those values were replaced by the mean over this 2-hour window. Static variables were repeated for each timestamp. The prediction target (a single aPTT measurement) is log-transformed during model training. The log transformation is discussed in the Results section. All model parameters are optimized on the mean-squared error (MSE) loss function. Additionally, we evaluated the mean absolute error and the explained variance for each model.

The 6 regression models were linear regression, elastic net, generalized linear model, support vector machine regression (SVR), K-nearest neighbor regression (KNN), and regression trees. We optimized hyperparameters using a grid search with 5-fold cross-validation. For the cross-validation, training and validation data were combined. The hyperparameter grids are shown in Table 1.

The models, cross-validation, and the grid search routine were from the scikit-learn package [24] and implemented in Python (The Python Software Foundation).

**Table 1:** Hyperparameters for each static model.

Model	Hyperparameters
Linear regression	None
Elastic net	α = (10^–4^, 10^–3^, 10^–2^, 10^–1^, 1, 2, 3) _L1_ratio = (0, 0.1, … 1.0)
GLM^a^	Power = (0, 1, 2, 3) α = (10^–2^, 10^–1^, 1, 2, 3)
SVR^b^	Kernel = (“linear,” “poly,” “rbf,” “sigmoid”) Degree = (2, 3, 4, 5, 6)
KNN^c^	K = (2, 3, 4, 5, 6, 7, 8, 9, 10) Weights = (“uniform,” “distance”)
Regression trees	Max_depth = (2, 3, 4, 5, unlimited) Min_samples_split = (2, 3, 4, 5, 6) Min_samples_leaf = (1, 2, 3, 4, 5)

^a^GLM: generalized linear model.

^b^SVR: support vector machine regression.

^c^KNN: K-nearest neighbor regression.

### Recurrent Neural Network Model

**Figure 1:**
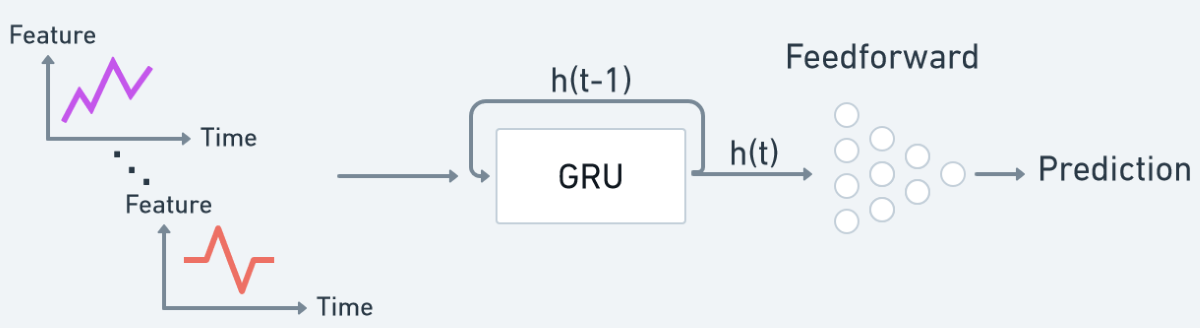
Schematic overview of input features, recurrent neural network, and feedforward network. GRU: gated recurrent unit.

This model consists of a gated recurrent unit (GRU), which can process a time series of arbitrary length and a fully connected network that uses the output of the GRU as input. Since we are only interested in predicting a single value, only the last output of the GRU is fed into a 3-layer fully connected model. No activation function is used between the output of the GRU and the first fully connected layer. The outputs of the 2 fully connected layers have rectified linear unit activation functions [25], and the final layer has no activation function. A schematic overview can be seen in Figure 1.

As for the previously described models, the recurrent neural network was optimized on the MSE. For experiments with the recurrent neural network, weights were optimized on the training set, and the results between experiments were compared on the validation set. We used the Adam optimizer with L2 penalty [26]. For each experiment, we chose weights with the lowest error on the validation set, which may occur before the maximum number of epochs are reached.

This model is significantly more costly to train compared to “static” models. Therefore, we did not perform a systematic hyperparameter optimization but ran several experiments with different hyperparameters and handpicked the best set of hyperparameters, which are shown in the Results section. Hyperparameters for the GRU submodel are hidden size (n=1, 2, 3, …), bidirectional connection (True, False), and the number of layers (n=1, 2, 3, …).

The 3-layered fully connected submodel had the number of neurons in each layer as 3 hyperparameters. Hyperparameters related to the training are the learning rate, L2 penalty, and the maximum number of epochs.

Patients have a different number of inputs per feature, since they receive their continuous heparin treatment at different times within their hospital stay. Thus, for training, we are limited to a batch size of 1 but accumulate multiple batches before weights are updated. To combat overfitting, we used an L2 penalty on the weights in the fully connected part of the model and chose weights on the epoch with the highest performance on the validation set.

All models and training scripts are available on github [27].

### Classification Models

To phrase aPTT prediction as a classification task, we used the 3 ranges first introduced by Ghassemi et al [14] of subtherapeutic for values below 60 s, therapeutic for values between 60 s to 100 s, and supratherapeutic for values above 100 s for the aPTT measurements. We compared our GRU model to the logistic regression model from Ghassemi et al [14] and the feedforward neural networks models by Su et al [15] and Li et al [18]. All parameters were taken from the reference literature for the respective model. For the feedforward networks from Su et al [15] and Li et al [18], we used cross-entropy [28] as a loss function with early stopping since the loss functions are not mentioned in the references.

The 3 classification models are retrained on the training split and receive the last value of each feature before the 12-hour cutoff in the same manner as the “static” regression models. The GRU is not retrained on the classification task, but the numeric predictions are binned into the 3 ranges post hoc. We evaluated the models on macroaveraged precision, macroaveraged recall, macroaveraged F_1_-score, and accuracy [29].

## Results

### Patient Cohort

**Figure 2:**
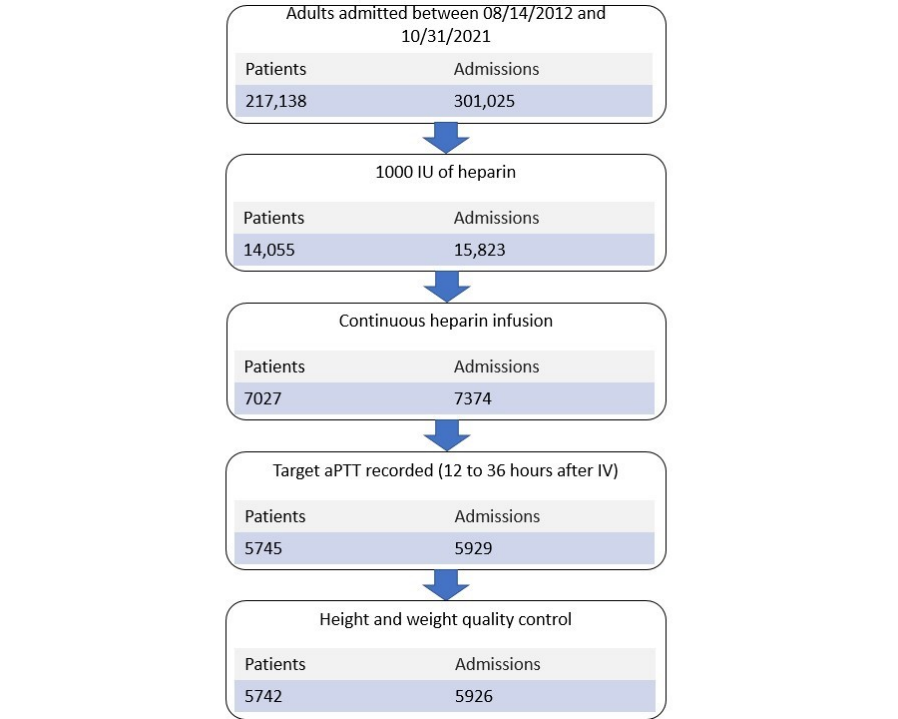
Flow diagram of unique patients and admissions that satisfy the specified inclusion criteria. aPTT: activated partial thromboplastin time; IV: intravenous line.

A flow diagram of consecutively applied filter criteria (specified in the methods section) to the entire patient cohort is shown in Figure 2. The selection criteria resulted in 5926 hospital admissions from a total of 5742 unique patients. Given that fewer than 4% of admissions occurred for previously admitted patients, we considered hospital admissions to be independent events. Basic patient characteristics and missing values are documented in Table 2.

Before model training or parameter estimation for mean and SD were performed, the admissions were split into training (n=3800), validation (n=945), and test (n=1181) samples. We ensured that different admissions by the same patient were in the same fold.

**Table 2:** Basic characteristics of the study cohort. The third column indicates how many patients do not have a single measurement during the hospital admission.

Feature		Value (N=5926)	Patients missing for entire stay, n (%)
Age (years), median (IQR)		70.62 (60.95-77.74)	0 (0)
**Gender, n (%)**			0 (0)
	Female	1910 (32)	N/A^a^
	Male	4016 (68)	N/A
Height (cm), median (IQR)		172 (164-178)	0 (0)
Weight (kg), median (IQR)		77 (66-90)	0 (0)
SOFA^b^, median (IQR)		5 (2-8)	442 (7.46)
SAPS II^c^, median (IQR)		36 (27-47)	449 (7.58)
APACHE II^d^, median (IQR)		17 (12-23)	525 (8.86)
TISS-10^e^, median (IQR)		10 (5-15)	5755 (97.11)
Dialysis, n (%)		449 (7.57)	0 (0)
ECMO^f^, n (%)		76 (1.28)	0 (0)
aPTT^g^ (s), median (IQR)		42.6 (36.1-54.6)	0 (0)
Bilirubin (mg/dL), median (IQR)		0.6 (0.35-1.24)	2529 (42.69)
CRP^h^ (mg/L), median (IQR)		56.2 (18.6-118.8)	1782 (30.07)
Gfr^i^ (count), median (IQR)		67 (39-90)	71 (1.20)
Creatinine (mg/dL), median (IQR)		1.01 (0.74-1.56)	32 (0.54)
Quick value (%), median (IQR)		76 (64-87)	17 (0.29)
Platelet count (per nL), median (IQR)		204 (139-292)	19 (0.32)
Total heparin administered (IU), median (IQR)		32398 (9500-90000)	0 (0)

^a^N/A: not applicable.

^b^SOFA: sequential organ failure assessment.

^c^SAPS II: simplified acute physiology score II.

^d^APACHE II: acute physiology and chronic health evaluation II.

^e^TISS-10: therapeutic intervention scoring system 10.

^f^ECMO: extracorporeal membrane oxygenation.

^g^aPTT: activated partial thromboplastin time.

^h^CRP: C-reactive protein.

^i^Gfr: glomerular filtration rate.

### Distribution of aPTT Values

**Figure 3:**
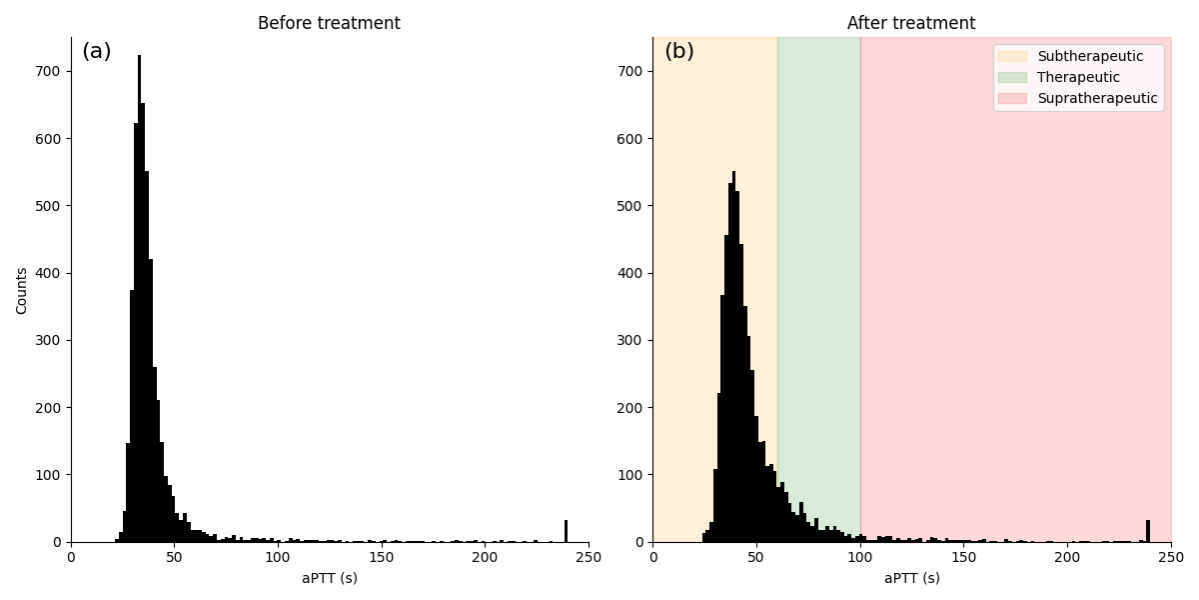
Histogram of aPTT values before treatment (a) and after treatment (b) with intravenous heparin. The histogram was obtained through binning, using 120 bins between minimal and maximal values. Shaded regions indicate regions identified in Ghassemi et al [14] and Su et al [15]. aPTT: activated partial thromboplastin time.

A histogram of measured aPTT before and after treatment is shown in Figure 3. In our cohort, both aPTT distributions before and after heparin treatment are narrowly peaked with a heavy tail. Values above 100 s occur very rarely. Small peaks are visible at 240 s where the laboratory reports some values as >240 s, which is mapped to 240 s.

The effect of heparin treatment on the entire cohort is clearly seen by the shift of the distribution. The difference in means is 8.64 s (95% CI 7.72-9.56; *P*<.001). The first 4 moments of the distribution of aPTT at *t*=0 and at *t*=24 are documented in Table 3. The mean aPTT value is higher after continuous heparin delivery compared to before treatment. Skew and kurtosis (while smaller after treatment) quantifiably indicate that the aPTT distribution is not symmetric and has a heavy tail. This fact makes the prediction of aPTT challenging. To make the learning task easier for our models, we log-transform the target variable to reduce skew and kurtosis. In effect, this makes “rare” events in the original distribution easier to predict.

The distribution that we observed in the Charité cohort contrasts with the aPTT values that are documented by other authors. Su et al [15] and Ghassemi et al [14] base their modeling studies on the Medical Information Mart for Intensive Care (MIMIC) II/III and eICU databases. The distribution of aPTT on the eICU database [15] is more heavy tailed than is the MIMIC cohort, however, less so than is our cohort. The 3 treatment categories reported in those works are indicated as shaded regions in Figure 3b. However, we do not classify our cohort into these categories but treat the prediction of aPTT after treatment as a regression problem.

**Table 3:** Statistical description of the binned distribution of aPTT values before continuous heparin treatment (*t*=0), 24 hours after continuous treatment commenced (*t*=24), and the log-transformed distribution after 24 hours.

	aPTT^a^ (t=0)	aPTT (*t*=24)	Log (aPTT [*t*=24])
Observations, n	4850	5926	5926
Mean	40.64	49.28	3.83
Variance	561.55	608.19	0.11
Skew	6.11	4.74	1.91
Kurtosis	42.93	26.71	5.37

^a^aPTT: activated partial thromboplastin time.

### Model Comparisons

In this section, the results of comparing 7 different models on the prediction of aPTT (see Table 4) are shown. Models 1-6 received only the last-measured values of each input feature before the 12-hour cutoff. We optimized hyperparameters for each model using a grid search and 5-fold cross-validation. The reported results are based on the test data that was not included in the 5 folds. A full description of the used grids appears in the Methods section. The best parameters for Models 1-6 are documented in Multimedia Appendix 1.

Model 7 (recurrent neural network) consumes the entire time series, resampled to 2-hour timestamps, for each input feature. We experimented also with resampling to 1-hour time steps and 4-hour time steps and found that the performance was similar (see Multimedia Appendix 1 for numerical results).

It is the most complex model in the comparison and ingests data from up to 7 days before continuous treatment to 12 hours after continuous treatment is administered. A systematic hyperparameter optimization for Model 7 was not performed; hence, we are underestimating the performance of the recurrent neural network in comparison to other models.

However, the recurrent neural network model achieved the highest score on the explained variance and MSE metrics. It ranked second to the SVR model on the mean absolute error (which penalizes outliers less than does the MSE). The SVR models ranked second to the recurrent neural network model on explained variance and MSE.

CIs were obtained by taking 1000 random samples of the same size as the test set, with replacement. Given that the distribution had a small number of large outliers, which had a significant effect on the quantity of interest, the CIs are wide.

**Table 4:** Comparison of different models for explained variance (higher is better), mean-squared error (lower is better), and mean absolute error (lower is better) obtained by resampling 1000 samples from the test set.

	Model	Explained variance	MSE^a^	MAE^b^
1	Linear regression, test set value, (95% CI)	0.163 (0.115-0.211)	0.487 (0.425-0.556)	0.474 (0.45-0.497)
2	Elastic net regression	0.168 (0.124-0.214)	0.484 (0.433-0.554)	0.474 (0.453-0.497)
3	GLM^c^	0.169 (0.121-0.21)	0.484 (0.422-0.556)	0.473 (0.450-0.5)
4	Support vector regression	0.203 (0.161-0.244)	0.476 (0.406-0.554)	0.442 (0.418-0.469)
5	Nearest neighbors	0.101 (0.055-0.140)	0.529 (0.460-0.597)	0.502 (0.477-0.528)
6	Decision tree regression	0.154 (0.108-0.198)	0.492 (0.427-0.563)	0.471 (0.447-0.495)
7	Recurrent NN^d^	0.21 (0.165-0.254)	0.459 (0.4-0.523)	0.454 (0.432-0.477)

^a^MSE: mean-squared error.

^b^MAE: mean absolute error.

^c^GLM: generalized linear model.

^d^NN: neural network.

### Prediction of aPTT by the Recurrent Neural Network Model

In this section we present the results of the recurrent neural network model and compare predictions with measured values on the test set. Multiple experiments with the model were performed, and the best handpicked parameters are shown in Table 5.

**Figure 4:**
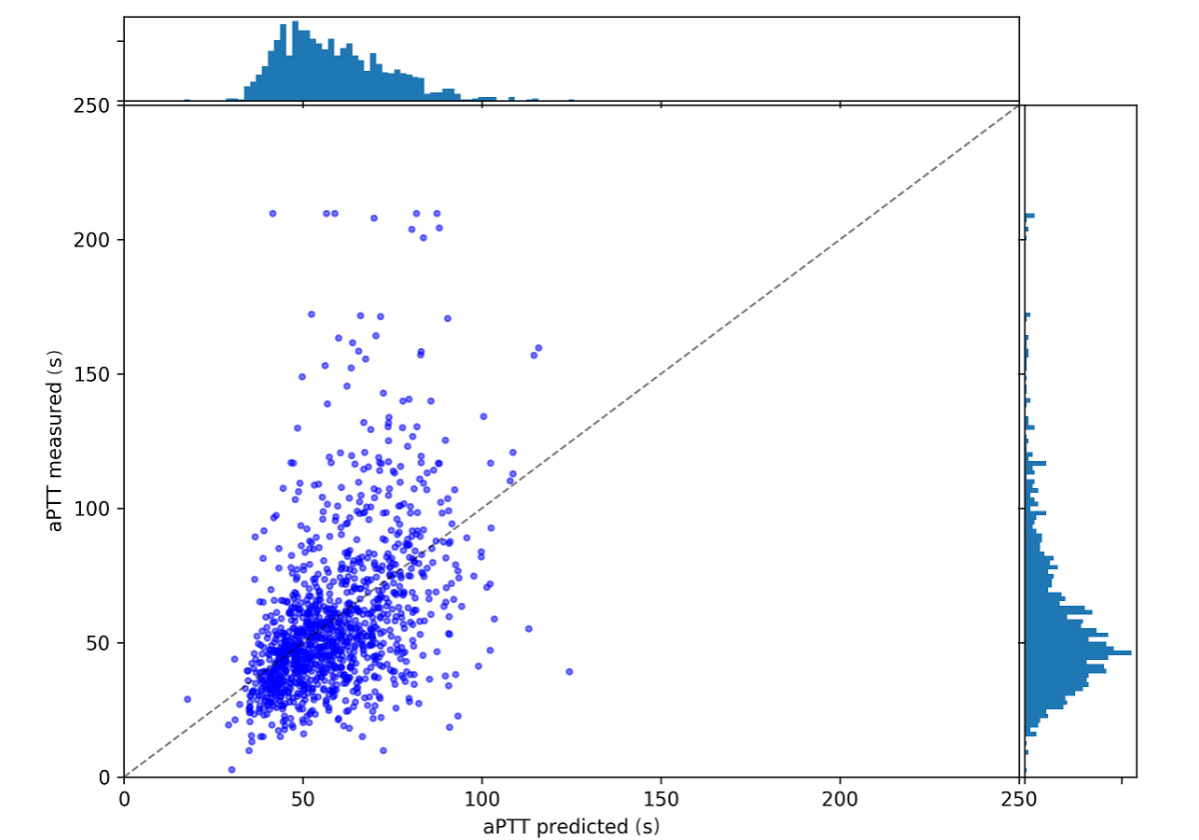
Predictions versus measurements. The figure shows predicted (abcissa) and measured aPTT (ordinate) after 24 hours in the central panel. Only predictions on the test set are shown. The dashed diagonal line indicates a perfect match between prediction and measurement. Above and to the right are binned distributions of all predictions and measurements, respectively. aPTT: activated partial thromboplastin time.

Predictions and measurements are shown in Figure 4. The distributions of aPTT values in the test data alone show a similar distribution as the aPTT values over the entire data set (cf Figure 3 and Figure 4 right panel). The histogram of predictions of the recurrent neural network model has a similar shape (cf Figure 4 top panel and Figure 4 right panel).

Direct comparisons between predictions and measurements can be seen in the center of Figure 4. The model can predict the majority of aPTT values very well. Although some outliers are predicted accurately, there are a few outliers above 150 s where predictions fall below 75 s. Likewise, some predicted outliers do not manifest as actual outliers.

**Table 5:** Best hyperparameters for the recurrent neural network model.

Parameter	Value
Learning rate	1e^–3^
Layers	Single GRU^a^ layer; 3 feedforward layers with 10, 5, and 1 output neurons, respectively
Hidden size (GRU)	5
Bidirectional	True
Accumulate gradient batches	16
L2 penalty on all weights	0.2

^a^GRU: gated recurrent unit.

### Comparison With Classification Models

In the previous sections, we have seen that the recurrent neural network shows the highest performance on the regression task. However, it is also apparent that not all predictions are accurate. To understand whether improvements needed to occur on the models or on data quality aspects, we rephrased the problem as a classification task to be able to compare the performance of the trained model with the 3 most recently published classification models [14,15,18]. Each of the 3 models was trained on our data set (details in the Methods section).

Our recurrent neural network scored the highest performance in recall and F_1_-score. The simplest model (logistic regression by Ghassemi et al [14]) had the highest precision, and the feedforward neural network by Li et al [18] had the highest accuracy (see Table 6 for results). No single model outperformed the others on all 4 metrics, and the appropriate model may be chosen depending on which metric is considered most relevant.

The fact that the best-published models show a comparable performance indicate that significant improvements require a closer monitoring of patients, additional tests, and improved data quality.

**Table 6:** Comparison of different models when formulating activated partial thromboplastin time prediction as a classification task. For each metric, a higher score is better.

Model	Precision	Recall	*F*_1_-score	Accuracy
GRU^a^	0.411	0.396	0.398	0.829
Ghassemi [14]	0.707	0.357	0.356	0.825
Su [15]	0.357	0.338	0.316	0.834
Li [18]	0.430	0.350	0.338	0.838

^a^GRU: gated recurrent unit.

## Discussion

### Principal Findings

In this study, we analyzed and predicted the effect of heparin treatment on a cohort of 5742 patients and 5926 hospital admissions 24 hours after continuous application. A statistically significant shift of aPTT measurements compared to the beginning of the treatment was observed. Most patients’ aPTT measurements were within 35 s to 75 s; however, some patients showed much higher aPTT values, leading to a challenging prediction problem with a long-tailed distribution. We demonstrated that ML models can aid in predicting the aPTT values 12 hours in advance. Additionally, we have shown that using the time series of variables improves predictive performance.

Some underlying medical conditions, while occurring rarely, are known to cause much higher aPTT values. These medical conditions include lupus anticoagulants or deficiencies in the intrinsic (deficiency in factors IX or X) or extrinsic pathways (deficiency in factors VII) [30,31]. These conditions are not routinely checked for and are only diagnosed when advanced lab testing is ordered.

Established guidelines aim for a prolongation of aPTT by 1.5 to 2.5 times [11-13]. Since patients have different aPTT values before heparin is administered, the target value according to the guidelines is different. Furthermore, medical professionals may define individual anticoagulation targets that do not match a prolongation of 1.5 to 2.5 times the baseline value. Thus, we consider aPTT prediction to be a regression problem as Kong et al [16] and Smith et al [17] have done. A model that predicts aPTT several hours before blood is drawn and analyzed can serve as a valuable aid in adjusting the heparin dosing to meet the patient’s aPTT target earlier.

In principle, aPTT can be predicted continuously. However, to allow a comparison between models that make a single prediction based on measurements at a single point in time and a model that consumes the entire time series, we fixed 2 time points (at 12 hours and 24 hours after continuous treatment started). Models can use data available at 12 hours and make a prediction for 24 hours after continuous treatment starts. The cutoff after 12 hours is arbitrary and could be reasonably made at a different time. The second point in time is motivated by the observation that reaching the aPTT target within 24 hours is associated with favorable outcomes [6]. The recurrent neural network showed the best performance, and its predictions were analyzed in detail. Although most samples were predicted well, an unsolved problem is that rare cases exhibit a remarkably high aPTT and are not captured by the model. As mentioned earlier, underlying medical conditions are known to cause significantly longer aPTT. We hypothesized that, for significantly improved predictions, either testing of conditions that cause a long aPTT or much more frequent measurements of aPTT combined with dosing adjustments are required.

Recent literature on aPTT prediction after heparin treatment considers 3 distinct ranges [14,15,18]. In order to compare our model to those in the literature, we binned our predictions into subtherapeutic, therapeutic, and supratherapeutic as introduced by Ghassemi et al [14]. We observed that our model showed a higher recall and F_1_-score than did the other models. Arguably, the setup that we chose was the most difficult compared to the references since we predicted a single aPTT value 12 to 36 hours in advance. Others made predictions 4 to 6 hours [15] or 4 to 8 hours [14] in advance or averaged aPTT measurements between 4 and 24 hours [18].

### Limitations

Other anticoagulants, such as warfarin or argatroban, were not considered. We expect that only a small sample of patients, if any, are receiving heparin together with anticoagulants and, therefore, decided not to take it into account as is common in similar studies [19].

It is well known that the laboratory conditions can affect the ranges of aPTT measurements [32]. The aPTT measurements were all reported by the same laboratory. Thus, the model may not be applicable to other centers and laboratories without parameter fine-tuning.

Modeling decisions that may negatively affect the model performance are the resampling of time series to 2-hour intervals. This resampling might miss significant changes in some variables. Furthermore, handling of missing data by forward and mean imputation could be improved by multiple imputation methods.

### Conclusions

Anticoagulation therapy with heparin monitored by the aPTT laboratory assay is a widely used procedure in ICUs. It is well known that heparin dosing is challenging due to high interpatient variability. In the future, ML may help to suggest personalized dosing recommendations. We demonstrated that a model based on time series performs best.

## Appendix

Multimedia Appendix 1 The best hyperparameters of the static models along with additional evaluation metrics.

## Abbreviations

APACHE II: acute physiology and chronic health evaluation II
aPTT: activated partial thromboplastin time
Charité: Charité – Universitätsmedizin Berlin
ECMO: extracorporeal membrane oxygenation
GRU: gated recurrent unit
ICU: intensive care unit
MIMIC: Multiparameter Intelligent Monitoring in Intensive Care
ML: machine learning
MSE: mean-squared error
SAPS II: simplified acute physiology score II
SOFA: sequential organ failure assessment
SVR: support vector machine regression
TISS-10: therapeutic intervention scoring system
