# Supplementary material for: A Recurrent Neural Network Model for Predicting Activated Partial Thromboplastin Time After Treatment With Heparin: Retrospective Study
Source: JMIR Med Inform. 2022 Oct 13;10(10):e39187. doi: 10.2196/39187 (PMC9614623; doi:10.2196/39187)
Supplement: Multimedia Appendix 1 [file medinform_v10i10e39187_app1.doc]

## Appendix

Table S1: Best Hyperparameters for each static model identified by grid search.

| Model | Best Hyperparameters |
| --- | --- |
| Elastic Net | α = 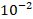  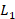ratio = 0.1 |
| GLM | power = 0  α = 10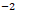 |
| SVR | kernel = “rbf”  degree = 2 |
| KNN | K = 10  weights = “uniform” |
| Regression Trees | max_depth = 3  min_samples_split = 6  min_samples_leaf = 4 |

Fig S1: Histograms of the three evaluated metrics (explained variance, mean-squared error and mean-absolute error) for the bootstrapped samples of the RNN and the SVR models.


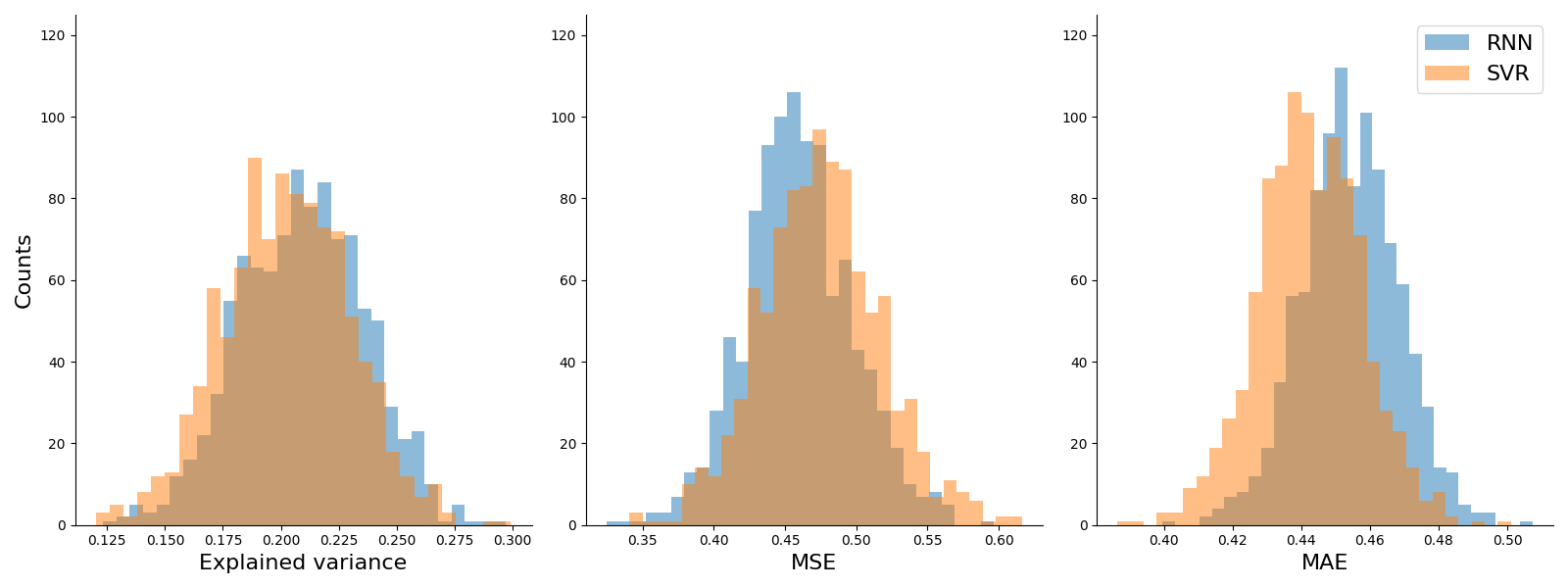


Table S2: Explained variance, mean-squared error and mean-absolute error for different resampling times.

|  | EV | MSE | MAE |
| --- | --- | --- | --- |
| 1H | 0.222  (0.195, 0.249) | 0.544  (0.432, 0.567) | 0.460  (0.456, 0.505) |
| 2H | 0.21  (0.165, 0.254) | 0.459  (0.400, 0.523) | 0.454  (0.432, 0.477) |
| 4H | 0.255  (0.216, 0.295) | 0.463  (0.409, 0.545) | 0.479  (0.437, 0.485) |
